# The value of survival analyses for evidence-based rural medical workforce planning

**DOI:** 10.1186/1478-4491-11-65

**Published:** 2013-12-11

**Authors:** Deborah J Russell, John S Humphreys, Matthew R McGrail, W Ian Cameron, Peter J Williams

**Affiliations:** 1 Centre of Research Excellence in Rural and Remote Primary Health Care, School of Rural Health, Monash University, PO Box 666, Bendigo, Victoria 3552, Australia; 2 Gippsland Medical School, Monash University, Northways Road, Churchill, Victoria 3842, Australia; 3 NSW Rural Doctors Network, Head Office, Suite 19, Level 3, 133 King Street, Newcastle, New South Wales 2300, Australia

**Keywords:** Australia, Cohort studies, Family physician, Family practice, General practitioner, Health manpower, Health policy, Health workforce, Personnel turnover, Policy making, Primary health care, Retention

## Abstract

**Background:**

Globally, abundant opportunities exist for policymakers to improve the accessibility of rural and remote populations to primary health care through improving workforce retention. This paper aims to identify and quantify the most important factors associated with rural and remote Australian family physician turnover, and to demonstrate how evidence generated by survival analysis of health workforce data can inform rural workforce policy making.

**Methods:**

A secondary analysis of longitudinal data collected by the New South Wales (NSW) Rural Doctors Network for all family physicians working in rural or remote NSW between January 1^st^ 2003 and December 31^st^ 2012 was performed. The Prentice, Williams and Peterson statistical model for survival analysis was used to identify and quantify risk factors for rural NSW family physician turnover.

**Results:**

Multivariate modelling revealed a higher (2.65-fold) risk of family physician turnover in small, remote locations compared to that in small closely settled locations. Family physicians who graduated from countries other than Australia, United Kingdom, United States of America, New Zealand, Ireland, and Canada also had a higher (1.45-fold) risk of turnover compared to Australian trained family physicians. This was after adjusting for the effects of conditional registration. Procedural skills and public hospital admitting rights were associated with a lower risk of turnover. These risks translate to a predicted median survival of 11 years for Australian-trained family physician non-proceduralists with hospital admitting rights working in small coastal closely settled locations compared to 3 years for family physicians in remote locations.

**Conclusions:**

This study provides rigorous empirical evidence of the strong association between population size and geographical location and the retention of family physicians in rural and remote NSW. This has important policy ramifications since retention grants for rural and remote family physicians in Australia are currently based on a geographical ‘remoteness’ classification rather than population size. In addition, this study demonstrates how survival analysis assists health workforce planning, such as through generating evidence to assist in benchmarking ‘reasonable’ lengths of practice in different geographic settings that might guide service obligation requirements.

## Background

Health workforce undersupply in rural areas is a persistent global problem, which contributes to inequitable health outcomes for rural populations in high-, middle-, and low-income countries alike [1-3]. Rural health workforce supply reflects the balance between current stocks and subsequent inflows (recruitment) and outflows (turnover) of workers. Considerable research has been undertaken into the complex range of issues that influence health workers’ decisions to take up, stay in, and leave rural practice (including economic, professional, personal, and community factors) [4-7].

Unfortunately, however, substantial gaps remain in our knowledge of the flows of health workers into and out of rural areas. Much of the existing research has focussed on health worker’s job satisfaction or intentions to leave rural practice, rather than on actual observed behaviour, though within the Australian rural context there are several exemplary studies [7,8]. Little is known, for example, about what length of stay might reasonably be predicted for a family physician practising in rural or remote locations [9]. This lack of empirical data on health worker flows and behaviours continues to hinder rural health workforce planning and decision making [10-12].

The research reported in this paper is designed to add to the existing evidence-base. The research aims first to identify and quantify the most important factors associated with the risk of rural and remote Australian family physicians leaving a practice, and secondly, to demonstrate the value of evidence generated by rigorous survival analysis of longitudinal health workforce data to inform rural health workforce planning and retention strategies. Although this paper examines the retention of family physicians within a single jurisdiction in one high-income country, there are important parallels with other similar geographically large developed countries, including Canada, United States of America, and Germany, and the analytical method demonstrated is one that can readily be adapted to a range of settings.

Improving our knowledge of what constitutes effective workforce retention strategies is dependent on several pre-requisites. In the first instance, it is important to understand what the most appropriate metrics are for measuring rural health worker turnover and retention. A second critical requirement for strengthening the evidence base is the availability of appropriate data and sufficient capacity to analyse and report selected indicators. Thirdly, the ability to make valid comparisons between different groups, and quantify differences in workforce retention is important for policy, as it assists targeting policy to specific groups of interest more effectively. A final requirement is familiarity with the types of interventions that might be used to improve retention, together with knowledge of their effectiveness and how much they might cost [13,14]. Within Australia, key current rural workforce strategies include the scaling of retention incentives according to location (based essentially on the degree of geographical remoteness) and restricting provider access to Medicare (the Australian universal health insurance scheme) for international doctors to designated ‘districts of workforce shortage’ and ‘areas of need’ [15]. However, despite the significant financial commitment to such programs, little is known about their effectiveness or their impact on patterns of turnover and retention. This paper seeks to show how empirically derived evidence can assist to inform policy development in this area.

## Methods

A recent review of the utility of different metrics for measuring health workforce turnover and retention in rural and remote contexts indicates that metrics derived using survival analysis methods have significant strengths to inform health workforce planning [16]. Survival analysis measures the time until an event occurs. In the case of this health workforce turnover and retention study, the event of interest is the time between take-up of a position until a health worker leaves that appointment. Hence, the data required include accurate commencement and exit dates for individual practitioners working in rural areas.

### Data

Despite the abundance of Australian medical workforce survey data collected over recent decades, health workforce planning and research is still handicapped by a lack of access to good national data at an individual practitioner level. For this study, rural and remote medical workforce data were available for the most populous Australian state, New South Wales (NSW). For more than ten years, longitudinal data have been collected by the NSW Rural Doctors Network (NSW RDN), a state and federally funded rural workforce agency established in 1998 to respond to workforce recruitment and retention issues facing rural family physicians in NSW. In Australia, family physicians are more commonly termed ‘general practitioners’ (GPs). Data are collected annually by the NSW RDN through a GP workforce and skills survey of GPs for the express purpose of rural workforce planning. These survey data are supplemented from other sources, including biannual practice manager surveys and the Australian Health Practitioner Regulation Agency register of physicians. Many of the data items are mandated as part of the National Minimum Data Set for rural health workforce agencies, which specifies core questions that have been developed and standardised across Australia’s states and territories [17].

**Figure 1:**
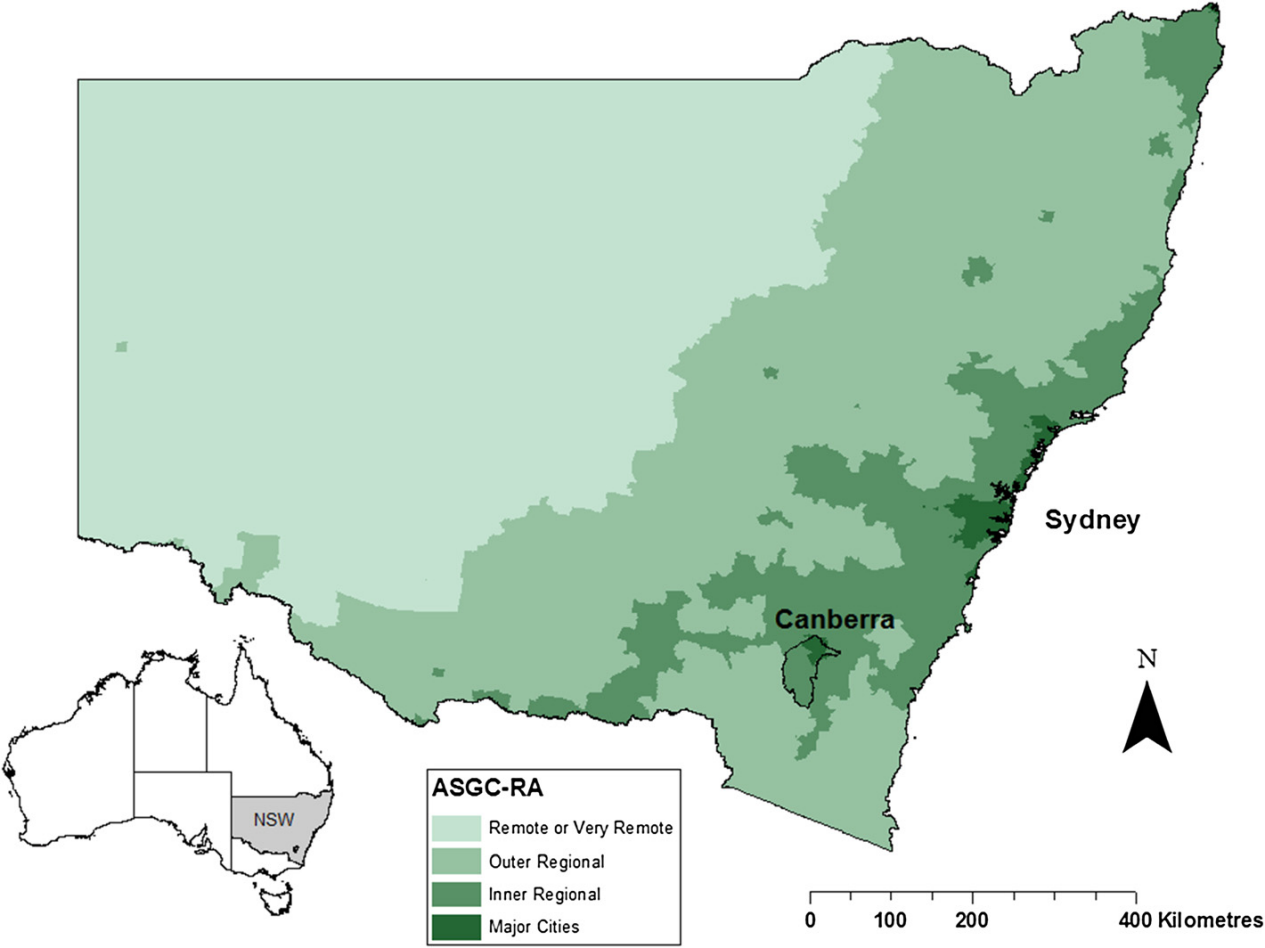
Map of the Australian standard geographical classification – remoteness areas for New South Wales, Australia.

Individual-level de-identified data were extracted for all family physicians who worked in non-metropolitan geographical locations in NSW at any time between January 1^st^ 2003 and December 31^st^ 2012. These include all inner regional, outer regional, remote, and very remote locations as defined by the Australian Standard Geographical Classification – Remoteness Areas (ASGC-RA) (Figure 1). The ASGC-RA classifies all of Australia based upon the road distance to the nearest city or town in each of five classes based on population size [18]. Data on community population sizes were obtained from the Australian Bureau of Statistics 2011 Census Urban Centres and Localities structure.

### Statistical analysis

The Kaplan-Meier method of survival analysis was used to analyse the data [19]. This technique enables employment data for all family physicians who have worked in rural NSW during the period of interest to be included in analysis. This is irrespective of whether or not they were practising in rural areas at the beginning of the study, or whether they were still providing primary care to rural NSW populations at the end of the study.

Each family physician could potentially have multiple ‘appointments’ over the 10-year period of the study. A new ‘appointment’ was defined each time a family physician moved their main practice location a distance of more than 15 km or had a break in continuous service provision of more than 3 months. A *‘*failure’ event was defined as a family physician leaving an appointment whilst a *‘*censored’ event was defined as a family physician remaining in an appointment at the end of the study observation period. Hence, another way of viewing a ‘failure’ is as a break in the provision of continuous care within a community. This was selected because relational continuity is known to be central to the development of trust and improved communication between doctors and their patients and to the securing of optimal health outcomes in the community [20]. Periods at risk were defined in days for each person.

Multiple ‘failures’ per family physician during the time period in question were permitted (though only one appointment could be held at a time), so the conditional risk set model proposed by Prentice, Williams and Peterson was used for modelling time until appointments ended [21]. This is an extension of the Cox proportional hazards model that stratifies by failure order and adjusts for violating the assumption of independence of failure times. The data were left truncated [22]; this meant that family physicians who already held an appointment at the start of the study were deemed to be ‘at risk’ of leaving that appointment only after January 1^st^ 2003. Main outcome measures were Cox proportional hazards ratios (comparative risk of one group of family physicians leaving an appointment compared to another group) and predicted median survival (the time in years, predicted by modelling, from commencing of appointments until half the workforce had left).

Family physician vocational trainees (or registrars) were excluded from analysis, as were family physicians in offshore locations (Lord Howe and Norfolk Islands), family physicians working in border towns located outside of NSW, and family physicians acting as *locum tenens*. Univariate analyses were initially undertaken and only independent variables with a *P* value less than 0.25 were tested in subsequent multivariate analyses. A stepwise elimination procedure was undertaken to derive the most parsimonious model, using a *P* value of 0.05 as the basis for elimination. In order to minimise listwise deletion occurring as a result of missing data, variables with 20% or more missing data were excluded from multivariate analysis.

Non-metropolitan communities were grouped by population size and geographical location guided by previous research which shows significant differentiation between them based on an association between family physician workload and town population size and geographical location [23,24]. Locations greater than 25 km from the coast were deemed to be inland.

Calculations were performed using StataIC, release 11.2 (StataCorp, College Station, TX, USA). Straight-line distances were calculated using ArcGIS 9.2 (ESRI, Redlands, CA, USA).

### Ethics approval

Ethics approval was received from the Monash University Human Research Ethics Committee (Ref. CF12/3902 – 2012001863).

## Results

Between January 1^st^ 2003 and December 31^st^ 2012 there were 3,354 family physician appointments in rural and remote NSW, representing 2,783 individual family physicians (83% of appointments were first rural appointments for that physician, 13% were second appointments, 3% were third appointments, and less than 1% were fourth or subsequent appointments). Over the 10-year period of this study, a total of 14,992 family physician-years of observation time were analysed, and 1,646 (49%) appointments ended – that is, on 49% of all occasions the doctor moved a distance greater than 15 km, or left the practice for a period of at least three months to undertake other activities. Of the 2,783 rural family physicians, 1,864 (67%) were male and 1,533 (55%) were known to be Australian graduates (Table 1).

**Table 1:** Characteristics of all family physicians who worked in rural NSW between 2003 and 2012

**Variable**	**Category**	**Frequency**	**Percent**
Gender	Male		1,864	67.0
	Female		919	33.0
Country of primary medical degree	Australia		1,533	55.1
	UK, Ireland, Canada, US, NZ		266	9.6
	Other		914	32.8
	Missing		70	2.5
Date of birth	Prior to 1950		463	16.6
	1950–1954		311	11.2
	1955–1959		396	14.2
	1960–1964		381	13.7
	1965–1969		386	13.9
	1970–1974		289	10.4
	During or after 1975		214	7.7
	Missing		343	12.3
Age at graduation	25 or younger		1,523	54.7
	Between 25 and 30		596	21.4
	30 or older		250	9.0
	Missing		414	14.9
	Total of each variable		2,783	100.0

Of the 3,354 family physician appointments in rural and remote NSW, 2,237 (67%) were known to be held by family physicians who were not undertaking procedural activities in any of anaesthetics, obstetrics, or operative surgery; 492 appointments (15%) were known to be associated with ‘conditional’ registration of the family physician (conditional registration in its various forms enables overseas trained doctors who are yet to gain Australian Medical Council accreditation to work in supervised practice in designated ‘districts of workforce shortage’ and ‘areas of need’ [15,25]); and 1,741 (52%) were known to be associated with the family physician having Visiting Medical Officer (VMO) rights (rights to provide medical services in a public hospital) (Table 2).

**Table 2:** Characteristics of family physician appointments in rural NSW between 2003 and 2012

**Variable**	**Category**	**Frequency**	**Percent**
Location population and remoteness	<5,000 population size and inner regional		582	17.4
	<5,000 population size and outer regional		433	12.9
	<5,000 population size and remote/very remote		92	2.7
	5,000–15,000 population size and inner regional		611	18.2
	≥5,000 population size and outer regional		277	8.3
	>15,000 population size and inner regional		1,359	40.5
Proceduralists	Yes		579	17.3
	No		2,237	66.7
	Missing		538	16.1
Registration	Full		2,656	79.2
	Conditional (Area of need or Overseas trained family physician)		492	14.7
	Missing		206	6.1
Visiting medical officer	Yes		1,741	51.9
	No		1,158	34.5
	Missing		455	13.6
	Total of each variable		3,354	100.0

**Figure 2:**
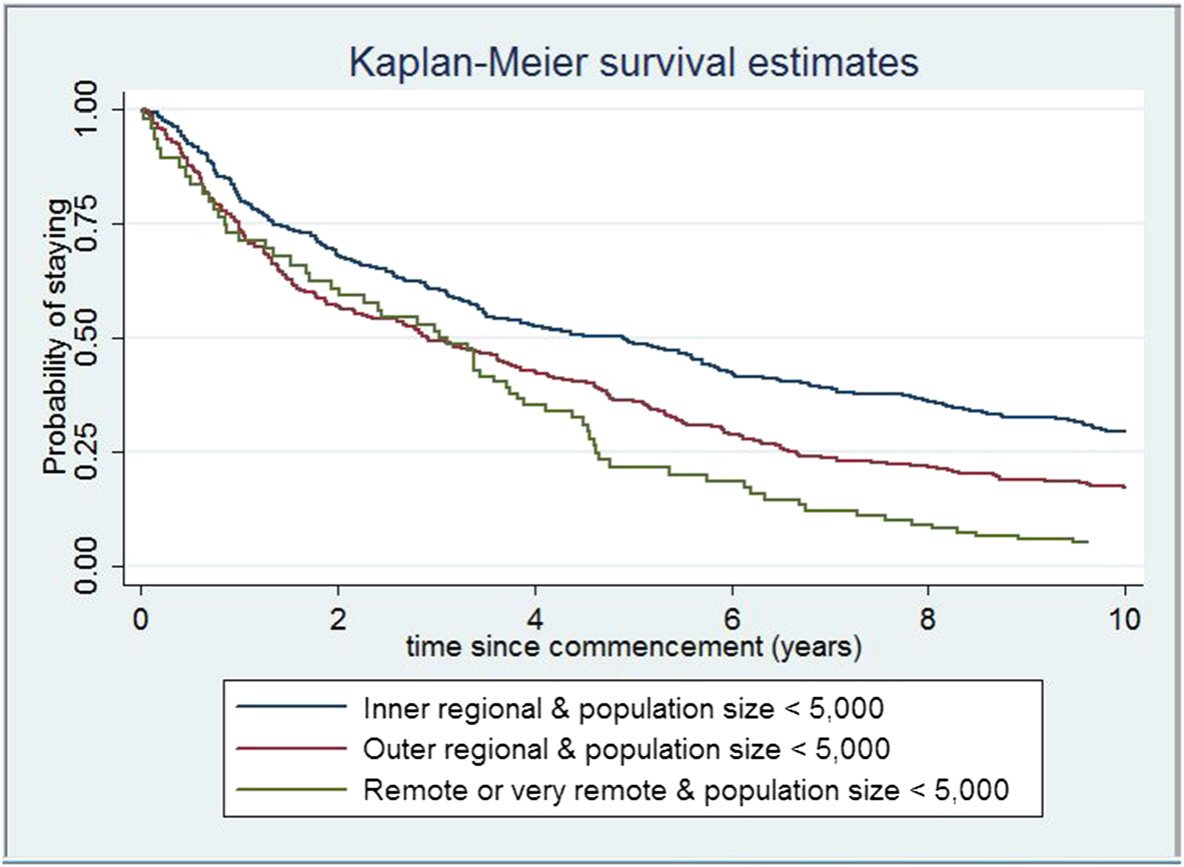
Family physician survival curves by geographic location and population size.

Univariate analyses revealed significant (α = 0.05) differences in the risk of family physicians leaving an appointment according to geographic location and population size, birth year, country in which the family physician obtained their medical degree, procedural skills, registration status, age at graduation, spousal rural location prior to the family physician’s first rural posting, and VMO rights, though not according to gender. Figure 2 illustrates retention patterns for family physicians working in small towns (population size <5,000) in inner regional, outer regional, and remote/very remote locations. Retention is higher in inner regional small towns compared with outer regional and remote/very remote small towns. Unadjusted estimates of the increased risk of leaving outer regional and remote/very remote small town family practices are 1.50 (1.25, 1.79) and 2.03 (1.61, 2.56) times the risk for inner regional small towns.

Multivariate Cox proportional hazards modelling revealed that family physicians practising in remote/very remote and outer regional towns with fewer than 5,000 inhabitants have a significantly increased risk of leaving town compared with family physicians located in inner regional NSW towns (Table 3). Family physician age was also significantly associated with risk of leaving town: the youngest family physicians (those born in 1970 or later) and the oldest family physicians (those born before 1945 and likely to be approaching retirement age) were at increased risk of leaving compared to family physicians born between 1945 and 1970; the increased risk for these groups was 1.54 and 1.45 times, respectively. Additional factors significantly associated with risk of family physicians leaving town include country of medical school graduation, procedural practice (not practising any of operative surgery, anaesthetics, or normal obstetric deliveries), having VMO rights, and holding conditional medical registration at any time during an appointment. The largest hazard ratio was for family physicians practising in small towns (population size <5,000) in remote/very remote Australia, and was associated with a 2.65 times greater risk of leaving compared to family physicians working in inner regional NSW. Graduating from medical schools in countries other than Australia, UK, Ireland, Canada, US, or New Zealand was associated with a 45% increased risk of leaving compared to the risk for Australian graduates. Family physician age upon graduation, however, was not significantly associated with turnover risk.

**Table 3:** Cox proportional hazards model: risk factors for rural family physicians leaving an appointment

**Variable**	**Baseline**	**Comparators**	**Hazard ratio**	**LL 95% CI**	**UL 95% CI**
Population size and remoteness classification	<5,000 and inner regional	<5,000 and outer regional	1.33	1.12	1.57
		<5,000 and remote/very remote	2.65	2.03	3.46
Country of primary medical degree	Australia	‘Other’ country	1.45	1.26	1.68
Proceduralist	Yes	No	1.42	1.21	1.68
Registration	Conditional	Full	1.49	1.24	1.79
Visiting medical officer rights	Yes	No	1.49	1.30	1.71
Birth year	1945–1970	Pre 1940	1.45	1.13	1.85
		1940–1945	1.36	1.03	1.79
		1970–1975	1.45	1.21	1.75
		After 1975	1.54	1.18	1.99
Coastal location	Yes	No	1.22	1.08	1.39

CI: Confidence interval; LL: Lower limit; UL: Upper limit; n = 2379 appointments after listwise deletion.

‘Other’ country – countries NOT including Australia, UK, Ireland, Canada, US, or New Zealand.

Translating these ratios into predictions of median survival (the length of time until half the workforce has left), revealed, for example, a difference in length of stay of 8.1 years on the basis of geography and population size alone for Australian-trained family physicians with VMO rights and not undertaking procedural activities. Predicted median survival for those working in small towns in coastal inner regional NSW was 11.1 years compared with 3.0 years in small towns in remote/very remote NSW (Table 4).

**Table 4:** Predicted median survival of rural family physicians based on Cox proportional hazards model

		**Predicted median survival (years)**^**†**^				
**Country of primary medical degree**	**Workload characteristics**	**Inner regional and population size less than 5,000**		**Outer regional and population size less than 5,000**		**Remote and population size less than 5000**
		**Coastal**	**Inland**	**Coastal**	**Inland**	**Inland**
Australia	Proceduralist	19.5	14.2	12.6	9.6	4.2
	VMO rights					
	Non-proceduralist	11.1	8.6	7.7	5.9	3.0
	VMO rights					
	Non-proceduralist	6.6	5.3	4.8	3.9	2.1
	No VMO rights					
‘Other’ country	Proceduralist	10.7	8.4	7.4	5.8	2.9
	VMO rights					
	Non-proceduralist	6.7	5.4	4.9	4.0	2.2
	VMO rights					
	Non-proceduralist	4.5	3.6	3.4	2.8	1.7
	No VMO rights					

^†^For fully registered family physicians born between 1945 and 1970; VMO: Visiting Medical Officer.

‘Other’ country – countries NOT including Australia, UK, Ireland, Canada, US, or New Zealand.

## Discussion

This innovative study breaks new ground in medical workforce research in Australia. For the first time, this study applies rigorous quantitative methods to Australian longitudinal medical workforce data to identify important correlates of the risk of family physicians leaving a rural or remote location. The use of survival (time to event) analysis enables important comparisons to be made on the basis of sentinel variables such as geographical location, population size, age, and professional status, and the statistical significance, magnitude, and direction of associations to be measured and reported. For the purpose of developing effective medical workforce policies and planning, these analyses provide several key insights.

First, our research shows that over the past ten years, the risk of family physicians leaving an appointment is strongly and significantly associated with geographical location and population size. For family physicians working in small towns with a population less than 5,000 a gradient of risk was found, whereby the risk of leaving was lowest in closely-settled coastal locations, intermediate in areas of moderate population density, and highest in the most sparsely-settled locations. For Australian trained family physicians who are non-procedural and have VMO rights, these findings translate into a predicted length of service of 11 years in small coastal towns in closely-settled locations. This compares with 6 years for family physicians in small inland towns with moderate population density and 3 years for family physicians in small inland towns in sparsely settled locations. Periods of service less than this might be interpreted as indicating ‘premature’ or ‘avoidable’ turnovers, and health authorities and workforce planning agencies could monitor any ‘hot spot’ locations to see whether any specific additional interventions are required in order to extend the length of practice of family physicians.

This significant differentiation in the risk of leaving is not surprising given the demonstration by Humphreys et al*.* of significant associations between professional indicators known to be related to family physician retention and geographical location and population size [23]. The extent to which shorter retention in small, more sparsely settled locations is ‘optimal’ (that is, all that might be expected in these locations) or ‘sub-optimal’ (that is, illustrates premature or avoidable turnover that could be adjusted through workforce incentives or interventions), remains a moot point. It is nevertheless important to interpret these observations in the context of substantial and increasing spending by the Australian federal government on direct financial incentives paid over this period to rural and remote family physicians in an attempt to improve retention. In particular, given that the Australian government is ‘scaling’ incentives according to geographical remoteness, these results provide, for the first time, empirical evidence to guide such differentiation in the allocation of retention incentives [25].

In Australia, expenditure on specific rural family physician workforce incentives has escalated almost six-fold from $19.9 million over the eight year period between 2004–2005 and 2012–2013 [25,26]. Whilst these incentives are scaled according to remoteness, community population size is not taken into account. In the absence of any definitive evidence about the effectiveness of medical workforce retention grants, our findings suggest that existing workforce retention interventions are insufficiently effective to ensure equality of continuity of family physician care for residents of remote and very remote areas. Given that numbers are small (92 appointments or less than 3% of total appointments) in remote and very remote NSW, opportunities exist to significantly strengthen retention strategies for this group of family physicians without necessarily having a large impact on the overall program budget. Improved targeting of retention strategies to family physicians in sparsely settled locations is especially pertinent given the recent finding that the major growth in family physician rural retention payments since 2010 has been in closely-settled areas, where retention is already relatively high [25].

In addition, the evidence generated by our study may help guide the relative lengths of service that might be required in the form of ‘return of service obligation’ for medical practitioners mandated or bonded to work in non-metropolitan areas. A range of Australian government programs currently scale return of service obligations, once again only according to geographic remoteness (ASGC-RA), but not on the basis of any empirical evidence. For example, the scaling ratio for reducing return of service obligations for the Medical Rural Bonded Scholarship Scheme is inner regional 1.0: outer regional 1.3: remote 1.5: very remote 1.8. Our work suggests that population size should also be taken into account and that the ratios for remote and very remote locations compared to inner regional locations could be higher.

A further important finding is that graduates from medical schools in countries without an Australian Medical Council-designated competent authority (countries other than Australia, UK, Canada, US, New Zealand and Ireland, which we term ‘other’ countries) had a substantially (1.45 times) increased risk of leaving a family physician appointment in rural NSW compared with Australian graduates. In terms of predicted median length of stay, this translates to Australian trained graduates staying for almost a year longer in small remote towns and for almost 2 years longer in small inland towns in regions of moderate population density. These differences are after the modelling adjusts for the lower risk of leaving an appointment for family physicians with conditional registration. Our findings are consistent with existing evidence that physicians obliged to work in a location not of their choosing are at increased risk of leaving that location in the longer term compared to non-obliged physicians [5,14]. These findings are particularly important for rural and remote workforce policy development since such a large proportion (33%) of family physicians in rural and remote NSW during the past 10 years are graduates of ‘other’ medical schools. Indeed, in 2009–2010 almost 50% of all family physicians in rural and remote Australia were international medical graduates [25]. Given this heavy reliance on internationally trained family physicians, it becomes critical to identify the root causes of their high turnover and address them as a matter of urgency. Recent work by McGrail et al. indicates a much higher relative dissatisfaction of overseas-trained family physicians (especially those with restrictions on where they can practise) compared with local graduates [27]. Their study also pinpoints various professional and non-professional aspects associated with dissatisfaction, some of which may be responsive to policy intervention.

Finally, our research has identified VMO status and procedural activities in the area of obstetrics, anaesthetics, and operative surgery as important correlates of reduced risk of family physician turnover, consistent with previous cross-sectional analyses [7]. Not only are these professional activities likely to be associated with a higher overall income, but also with a greater sense of autonomy, a wider variety of work, increased opportunities to use an extended skill set, and a heightened sense of responsibility. In other words, VMO status and procedural activity are associated with important indicators of family physician professional satisfaction [28], which may in turn be associated with reduced turnover. These findings have important implications for future investment in rural training pathways that develop advanced skills needed for rural and remote hospital work, as for instance with the successful generalist model being promoted in Queensland, Australia [25]. Furthermore, provision and maintenance of infrastructure to foster hospital-based activities of rural and remote family physicians is also important because of its association with relatively higher family physician retention.

A number of limitations of this study are acknowledged. First, despite family physician vocational trainees making a substantial and important contribution to the rural and remote workforce in NSW, they were excluded from analysis since the factors driving their relocation decisions are likely to be different from those of family physicians and because their training program often requires rotations at various times regardless of their satisfaction with any practice location. Second, some variables of interest were not included in the final multivariate model because of the extent of missing data. These included rural origin of spouse and recipient status for some specific rural scholarships. Some variables of current policy relevance, such as Medical Rural Bonded Scholarships, also had insufficient numbers of recipients to permit reliable estimation of the effect. This is due to the long lag time between receipt of financial support and commencement of rural or remote practice as a family physician. Third, the retention profile used in this study coded a ‘failure’ as a location move of more than 15 km or break in service provision of more than 3 months [29], in recognition of the importance of continuity of care as a key dimension of primary health care [30]. While realistic and relevant for our research in non-metropolitan Australia, this retention profile may not be appropriate for all workforce-planning purposes. Pathman’s pioneering work on physician retention described how physicians may variously be considered as being successfully retained when they stay with an initial practice, when they stay within the initial community, when they stay within any rural location within the jurisdiction, or even when they remain actively practising clinical medicine [29]. Indeed, a strength of survival analysis is that it can successfully be applied to each of these different policy problems simply by coding a ‘failure’ in different ways – for example, as leaving a particular ASGC-RA, or as leaving rural NSW or even as leaving the medical profession – depending on the availability of requisite data and the particular policy question being asked.

## Conclusions

This study highlights how survival analyses can be used to generate rigorous evidence to inform policy development in the area of health workforce planning, particularly, for example, in the strengthening and improved targeting of retention strategies in rural and remote areas. In this instance, survival analyses identified strong associations between geographical location and population size, country of primary medical degree, procedural activity, and VMO status, and the risk of NSW rural and remote family physicians leaving a community. Such quantitative empirical evidence establishes a better baseline against which to monitor the effectiveness of workforce strategies and guide workforce planning.

Importantly, the value of these analyses is their potential application across a wide range of countries, most notably high-income developed nations where workforce patterns and problems are not dissimilar to Australia. It is worth pointing out, however, that developing countries and even some rural and remote areas within developed countries may not have sufficiently supported human resource capacity to collect the required high quality data and undertake appropriate analyses without assistance from the regional offices of the health authorities responsible for human resource planning. The value of survival analyses is also applicable across a range of different health worker professions since both data collection and the method itself can be tailored to specific contexts. Development of empirical evidence in this way provides a far better basis than *ad hoc* cross-sectional turnover studies or anecdotal information to guide the development and evaluation of sound and comprehensive workforce retention strategies.

## Abbreviations

ASGC, RA: Australian standard geographical classification – remoteness areas; GP: General practitioner; NSW: New South Wales; RDN: Rural doctors Network; VMO: Visiting medical officer

## Competing interests

The authors declare that they have no competing interests.

## Authors’ contributions

DR and JH conceived and designed the study. IC and PW were responsible for the acquisition of data. DR, JH, and MMcG were involved in analysis and interpretation of the data. All authors were involved in drafting and critical revision of the manuscript. All authors read and approved the final manuscript.

## Authors’ information

DR, JH, and MM are members of the Centre of Research Excellence in Rural and Remote Primary Health Care (CRERRPHC), conducting research in accessible and equitable primary health service provision in rural and remote Australia. IC is the Chief Executive Officer and PW is the information manager at NSW RDN and IC is also a member of the National Advisory Committee of the CRERRPHC.
